# Effect of carbamazepine on dolutegravir pharmacokinetics and dosing recommendation

**DOI:** 10.1007/s00228-016-2020-6

**Published:** 2016-02-22

**Authors:** Ivy Song, Steve Weller, Juhin Patel, Julie Borland, Brian Wynne, Mike Choukour, Fred Jerva, Stephen Piscitelli

**Affiliations:** GlaxoSmithKline, Research Triangle Park, Durham, NC USA

**Keywords:** Carbamazepine, Dolutegravir, Drug interaction, Healthy subjects

## Abstract

**Purpose:**

Dolutegravir (DTG) is primarily metabolized by UGT1A1 with CYP3A as a minor route. Carbamazepine (CBZ) is a potent inducer of these enzymes; thus, the effect of oral extended-release CBZ on DTG pharmacokinetics (PK) was evaluated to provide dose recommendation when co-administered.

**Methods:**

This was a single-center, open-label, fixed-sequence, crossover study in healthy adults. Subjects received three treatments: DTG 50 mg every 24 h (q24h) × 5 days in period 1, followed by CBZ 100 mg every 12 h (q12h) × 3 days, then 200 mg q12h × 3 days, then 300 mg q12h × 10 days in period 2, and DTG 50 mg q24h + CBZ 300 mg q12h × 5 days in period 3. No washout intervals occurred. Each dose was administered with a moderate-fat meal. Serial PK samples for DTG were collected on day 5 of periods 1 and 3. Plasma DTG PK parameters were determined with non-compartmental analysis. Geometric least-squares mean ratios (GMRs) and 90 % confidence intervals (CIs) were generated by the mixed-effect model for within-subject treatment comparisons. Safety assessments were performed throughout the study.

**Results:**

Sixteen subjects enrolled; 14 completed the study. CBZ significantly reduced DTG exposure: GMRs (90 % CI) for DTG + CBZ versus DTG alone were 0.51 (0.48–0.549), 0.67 (0.61–0.73), and 0.27 (0.24–0.31) for area under the curve from time zero to the end of the dosing interval (AUC(0-τ)), maximum observed plasma concentration (Cmax), and plasma concentration at the end of the dosing interval (Cτ), respectively. DTG alone and co-administered with CBZ was well tolerated.

**Conclusion:**

Integrase strand transfer inhibitor-naive subjects taking CBZ should receive DTG 50 mg twice daily versus once daily, as is recommended with other potent UGT1A/CYP3A inducers.

ClinicalTrials.gov: NCT01967771

## Introduction

Dolutegravir (DTG, Tivicay^®^, ViiV Healthcare) is an HIV integrase strand transfer inhibitor approved for use in combination with other antiretrovirals for the treatment of HIV infection in adults and adolescents. The usual dose in integrase strand transfer inhibitor-naive subjects is 50 mg once daily [1]. Dolutegravir is a substrate of P-glycoprotein (P-gp) and is primarily metabolized by uridine diphosphate glucuronosyltransferase (UGT) 1A1 with a minor component (approximately 10 %) via cytochrome P450 (CYP) 3A4 [1, 2]. Clinically significant drug interactions requiring a dose adjustment with DTG have not been observed with inhibitors of UGT1A1, CYP3A4, or P-gp [3, 4]. However, a clinically significant decrease in exposure has been observed with DTG when co-administered with the strong CYP3A4 inducers that include tipranavir/ritonavir, efavirenz, and rifampin [5, 6]. These enzyme inducers require the dose of DTG to be increased from 50 mg once daily to 50 mg twice daily.

Neurologic manifestations of HIV infection are quite diverse, and many individuals with HIV infection who are taking antiretroviral therapy require treatment with anticonvulsants. Anticonvulsant medications are commonly used in patients with HIV either for seizure management or for expanded indications such as neuropathic pain and psychiatric disorders, including depression [7, 8]. Carbamazepine (CBZ) is an antiepileptic drug (AED) that has been used for the treatment of depression as well as trigeminal neuralgia. Carbamazepine is a potent enzyme inducer and is known to induce its own metabolism, resulting in a shorter half-life (12–17 h) and lower exposure following chronic dosing compared with the half-life (25–65 h) and exposure following single dosing [11]. In addition, potent induction of CYP3A4 and UGT1A1 enzymes by CBZ can lead to underexposure of drugs that are metabolized by these enzymes [9, 10]. Considering the metabolic pathways of DTG and CBZ and that CBZ may be prescribed to HIV-infected subjects, an evaluation of the effects of CBZ on DTG pharmacokinetics (PK) was warranted.

In vitro, DTG demonstrated minimal or no direct inhibition of CYP isozymes, UGT1A1, UGT2B7, and many transporters (P-gp, breast cancer resistance protein, OATP1B1, OATP1B3, MRP2) and was not an inducer of CYP1A2, CYP2B6, or CYP3A4 [1, 2]. Dolutegravir also had no significant effect on midazolam exposure, a probe substrate for CYP3A4, in healthy subjects [1]. Carbamazepine is primarily metabolized in the liver by CYP3A. However, based on DTG enzyme interaction profile, it was not expected to affect CBZ PK; thus, the primary objective of this study was to evaluate the effect of CBZ on DTG PK in healthy subjects and not vice versa.

## Methods

### Study design

This was a phase I, single-site, open-label, fixed-sequence, crossover study with three treatment periods to evaluate the effect of an oral extended-release formulation of CBZ on the steady-state PK of orally administered DTG in 16 healthy subjects. The study was conducted in accordance with the principles of the Declaration of Helsinki. A written informed consent was obtained from all subjects, and the protocol was approved by the institutional review board of the study site. The trial was registered at ClinicalTrials.gov (NCT01967771; protocol number: 200901).

Each subject was evaluated with physical examination, medical history, and laboratory testing at a screening visit conducted within 30 days prior to the first dose of drug and at a follow-up visit 7 to 14 days after the last dose of study drug. Eligible subjects were 18 to 65 years of age, had a minimum weight requirement of 45 kg for women and 50 kg for men, and a body mass index requirement of between 18.5 and 31.0 kg/m^2^. Subjects with positive pre-study drug/alcohol screens, positive HIV antibody test results, and positive serum or urine human chorionic gonadotropin test results for pregnancy, as well as Asians with positive HLA-B*1502 allele (carriage is associated with a risk of hypersensitivity reactions with exposure to CBZ) and subjects with allergies to tricyclic antidepressants, were excluded. Women of child-bearing potential agreed to protocol-specified methods of contraception to avoid pregnancy. Subjects were not allowed to receive prescription or non-prescription/herbal medications within 7 days of dosing or throughout the study. Use of antacids, vitamins, or calcium or iron supplements, which are known to reduce DTG absorption, was strictly prohibited within 24 h before the first dose of study medication and throughout the trial, including follow-up. Acetaminophen was permitted with a dose that did not exceed 2 g per day.

During period 1, subjects received DTG 50 mg once daily as a single agent for days 1 to 5. During period 2, subjects received CBZ alone and the dose was escalated: 100 mg twice daily for days 1 to 3, 200 mg twice daily for days 4 to 6, and 300 mg twice daily for days 7 to 16. During period 3, DTG 50 mg once daily was co-administered with CBZ 300 mg twice daily for days 1 to 5. There was no washout between treatment periods. As CBZ is recommended to be given with food per product information [11], all doses in the testing unit, including those on PK collection days, were administered orally in the morning 30 min after the start of the moderate-fat meal, and subjects were asked to do the same when dosing outside the unit. Subjects were housed at the clinical research unit from period 1, day 1, until the morning of period 2, day 8, as well as from period 2, day 16, through period 3, day 6. Phone call reminders and pill counts were employed when subjects self-administered CBZ as outpatients during period 2, days 8 to 16. Serial blood samples for determining the plasma concentration of DTG were collected on day 5 of period 1 and day 16 of period 3 at the following times: pre-dose (within 15 min before study dose), 0.5, 1, 2, 3, 4, 6, 12, and 24 h after DTG dosing.

The DTG dose selected for this study was the 50 mg once-daily dose regimen recommended for the treatment of HIV integrase inhibitor (INI)-naive subjects. A higher dose of 50 mg twice daily is recommended for subjects with INI resistance; however, this population represents a small portion of HIV-infected subjects to be treated with DTG. Therefore, the more common once-daily dose was evaluated in this study. Carbamazepine dose selection was based on the range of likely CBZ clinical doses [11], the time course and magnitude of CBZ induction effect observed in other studies [10, 12], and consideration of the risks/benefits of CBZ administration to healthy subjects [11]. Review of prior CBZ drug-interaction studies found most CYP3A/UGT1A1 substrate drugs demonstrated substantially altered PK when CBZ was dosed between 200 and 600 mg daily for 3 weeks or less. Carbamazepine is titrated to achieve seizure or pain control, with clinically effective (adult) doses ranging from 400 mg up to a maximum of 1000 mg daily. There is no benefit, and only theoretical risk, to dosing volunteers with a central nervous system-active drug such as CBZ. For this reason, in this study, CBZ was titrated upward over a 7-day period to a mid-range value of 600 mg daily, for a total of 21 days of CBZ exposure, to allow for complete or near complete induction. The drug-interaction PK assessment was obtained on the last day of period 3 (day 21).

### Safety evaluations

Safety evaluations included the monitoring of adverse events (AEs), physical examinations, clinical chemistry laboratory tests, vital signs, and electrocardiograms. Additionally, assessment of Columbia suicide severity rating scale scores was performed throughout the study, because CBZ exposure (along with exposure to several other AEDs) has been associated with increased risk of suicidal ideation and depression.

### Bioanalytical methods

Plasma samples were analyzed for DTG by PPD, Inc. (Middleton, WI) using a validated analytical method based on protein precipitation with acetonitrile containing the stable isotope-labeled internal standard (GSK1349572-d_7-_^15^N), followed by high-performance liquid chromatography/tandem mass spectrometry analysis. The lower limit of quantification for DTG in plasma was 20 ng/mL, and the upper limit of quantification was 20,000 ng/mL. Precision and accuracy were evaluated by replicate analyses of human plasma quality control samples prepared at five concentrations: 60, 160, 640, 2400, and 15,200 ng/mL. Precision, measured as the percent coefficient of variation, ranged from 3.0 to 5.9 % across the quality control range. Accuracy, expressed as the percent difference from the mean value, ranged from −2.2 to 1.8 %. Both were within acceptance standards of 15 %.

### Pharmacokinetic analysis

A non-compartmental PK analysis of the DTG concentration-time data was conducted by Phast Clinical Data, Inc. (Raleigh, NC). Pharmacokinetic analyses of plasma DTG concentration-time data were analyzed using Model 200 (for extravascular administration) of Phoenix WinNonlin version 6.3 (Pharsight Corporation, St. Louis, MO). Plasma PK parameters for DTG were calculated using actual elapsed times from dosing. The individual PK parameters that were determined included area under the curve from time zero to the end of the dosing interval (AUC(0-τ)), maximum observed plasma concentration (Cmax), plasma concentration at the end of the dosing interval (Cτ), oral clearance, and terminal half-life.

### Statistical analysis

This study was designed to estimate the magnitude of drug-interaction effect of CBZ on the PK parameters of DTG. No formal hypothesis was tested. Instead, an estimation approach was used to evaluate the effect of CBZ on DTG PK. Pharmacokinetic parameters were log-transformed and analyzed by analysis of variance to determine the point estimate and associated 90 % confidence intervals for the difference between test treatment (DTG + CBZ) and reference treatment (DTG alone). These values were then back-transformed to calculate the point and interval estimates for test-to-reference treatment ratios on the original scale. Geometric least-squares mean ratios and 90 % confidence intervals were generated by the mixed-effect model for within-subject treatment comparisons.

## Results

### Demographics

Sixteen subjects, 2 females and 14 males, were enrolled and included in the safety and PK populations. Two subjects were prematurely discontinued from the study: one male subject withdrew due to pyrexia/febrile illness on period 2, day 3 (CBZ alone), and one female subject withdrew due to drug-induced hypersensitivity syndrome, increased alanine aminotransferase (ALT) level, thrombocytopenia, and maculopapular rash in period 2, day 11 (CBZ alone). The mean (standard deviation (SD)) age was 38.6 (12.8) years and ranged between 18 and 65 years. The mean (SD) body mass index was 26.1 (3.2) kg/m^2^, height was 177.8 (6.3) cm, and weight was 82.4 (11.5) kg. Fifteen subjects (94 %) were not Hispanic or Latino. Six subjects (38 %) were African American or of African heritage, while 10 subjects (63 %) were White/Caucasian or of European heritage.

### Safety

No subjects experienced AEs during period 1 (DTG alone). Seven subjects experienced at least one AE: six subjects while taking CBZ alone and one subject in the DTG + CBZ group (Table 1). One subject in the DTG + CBZ group experienced a photosensitivity event to overhead fluorescent light that occurred during period 3 and resolved without interruption of drug administration within 5 days. Headache, thrombocytopenia, and nausea were the only AEs that occurred in more than one subject. No grade 3 or grade 4 AEs, deaths, or non-fatal serious AEs were reported. Two subjects were permanently discontinued because of AEs in period 2 (CBZ alone). One male subject developed a febrile illness after receiving CBZ in period 2. His lymphocyte count dropped to 0.5 × 10^−3^ cells/mm^3^, and ALT increased to 66 IU/L (grade 1), which was thought to be an unrelated viral infection. The second subject was a 23-year-old Caucasian female who developed a suspected moderate (grade 2) hypersensitivity syndrome that manifested with a facial rash and headache while she was receiving 300 mg CBZ twice daily during period 2. Laboratory analysis revealed a decrease in white blood cell count to 3800 cells/mm^3^ (compared with 8800 cells/mm^3^ at baseline), a decrease in platelet count to a low of 78,000 cells/mm^3^ (from 159,000 3 days before), and an increase in ALT level to a high of 151 IU/L (grade 2), while bilirubin levels remained within normal limits. This was considered possibly related to CBZ. The rash slowly resolved over the next 2 weeks, and all laboratory abnormalities in this subject resolved within 3 to 4 weeks of CBZ discontinuation.

**Table 1:** Summary of drug-related adverse events by treatment

	DTG alone (*n* = 16) *n* (%)	CBZ alone (*n* = 16) *n* (%)	DTG + CBZ (*n* = 14) *n* (%)
Any event	0	6 (38)	1 (7)
Asthenia	0	1 (6)	0
Fatigue	0	0	1 (7)
Pyrexia	0	1 (6)	0
Thirst	0	1 (6)	0
Headache	0	2 (13)	0
Cognitive disorder	0	1 (6)	0
Thrombocytopenia	0	2 (13)	0
Leukopenia	0	1 (6)	0
Lymphopenia	0	1 (6)	0
Drug reaction with eosinophilia and systemic symptoms	0	1 (6)	0
Photosensitivity reaction	0	0	1 (7)
Rash maculopapular	0	1 (6)	0
Nausea	0	1 (6)	0
ALT increased	0	1 (6)	0
Hyponatremia	0	1 (6)	0
Pain in extremity	0	1 (6)	0

Treatments: DTG alone = DTG 50 mg once daily (period 1, days 1–5); CBZ = CBZ 100 mg twice daily (period 2, days 1–3) + CBZ 200 mg twice daily (period 2, days 4–6) + CBZ 300 mg twice daily (period 2, days 7–16); DTG + CBZ = DTG 50 mg once daily + CBZ 300 mg twice daily (period 3, days 1–5)

*ALT* alanine aminotransferase, *CBZ* carbamazepine, *DTG* dolutegravir

### Pharmacokinetics

**Fig. 1:**
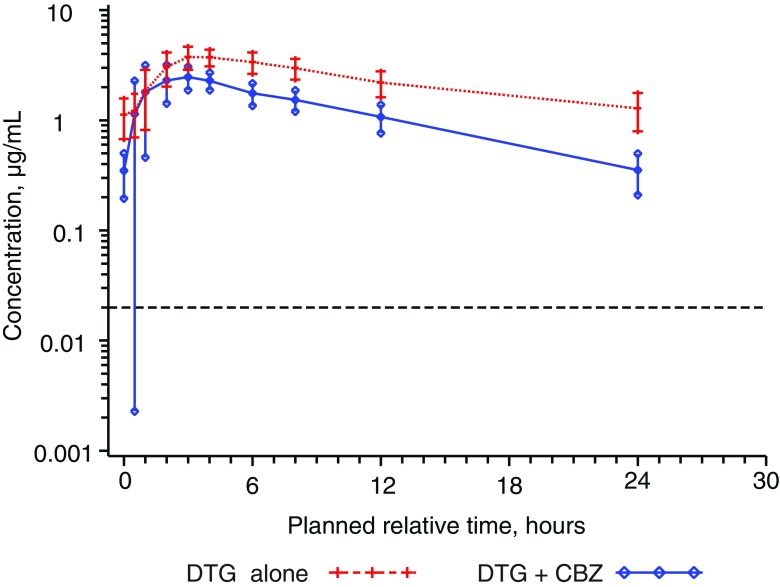
Mean ± SD concentration-time profile for dolutegravir (*DTG*) with and without concomitant administration of carbamazepine (*CBZ*). Treatment: DTG alone = DTG 50 mg once daily; DTG + CBZ = DTG 50 mg once daily + CBZ 300 mg twice daily

Pharmacokinetic parameters following repeat-dose administration of DTG with and without CBZ and treatment comparisons are shown in Table 2, and the mean (SD) concentration-time profiles are shown in Fig. 1. The geometric mean AUC(0-τ), Cmax, and Cτ of DTG were reduced by 49, 33, and 73 %, respectively, when DTG was co-administered with CBZ. Dolutegravir clearance was increased by 95 %, and its half-life shortened from 12.9 to 7.31 h when it was co-administered with CBZ. On an individual subject basis, the reduction in AUC(0-τ) ranged from 27 to 58 %, and the reduction in Cτ ranged from 53 to 82 % for the 14 subjects with PK results during both treatment periods.

**Table 2:** Summary and statistical comparison of plasma DTG PK parameters following DTG 50 mg once-daily dose administration with and without CBZ

PK parameter	Geometric mean (CV%)		Ratio of GLS means (90 % CI)
	DTG alone (*n* = 16)	DTG + CBZ (*n* = 14)	DTG + CBZ vs DTG alone
AUC(0-τ) (μg·h/mL)	53.8 (21.4)	27.4 (22.1)	0.512 (0.477, 0.549)
Cmax (μg/mL)	4.16 (14.4)	2.77 (25.0)	0.666 (0.610, 0.726)
Cτ (μg/mL)	1.20 (39.1)	0.325 (45.9)	0.274 (0.240, 0.313)
CL/F (L/h)	0.929 (21.4)	1.83 (22.1)	1.95 (1.82, 2.10)
t1/2 (h)	12.9 (23.8)	7.31 (16.9)	0.567 (0.526, 0.611)

Treatments: DTG alone = DTG 50 mg once daily; DTG + CBZ = DTG 50 mg once daily + CBZ 300 mg twice daily

*AUC*(*0-τ*) area under the curve from time zero to the end of the dosing interval, *Cτ* concentration at the end of the dosing interval, *CBZ* carbamazepine, *CI* confidence interval, *CL*/*F* oral clearance, *Cmax* maximum observed plasma concentration, *CV*% coefficient of variation, *DTG* dolutegravir, *GLS* geometric least squares, *PK* pharmacokinetic, *t1*/*2* terminal elimination half-life

## Discussion

Carbamazepine is a potent inducer of CYP3A4 and UGT1A1 enzymes and has been shown to decrease plasma concentrations of a number of drugs [9–13]. This study demonstrated that repeated dosing of CBZ for 21 days resulted in significant reductions of 49, 33, and 73 % in AUC(0-τ), Cmax, and Cτ, respectively, of DTG. Dolutegravir is primarily metabolized to an inactive ether glucuronide by UGT1A1, with some minor oxidative metabolism by CYP3A4; therefore, the increased DTG clearance observed in this study is likely a result of concurrent UGT1A1 and CYP3A4 induction by CBZ. Although regulatory guidance generally recommends that the highest possible clinical dose be used in the evaluation of a drug as a perpetrator of drug interactions, a lower but more commonly administered mid-range CBZ dose regimen (titration up to 600 mg/day) was selected for this study to minimize adverse effects in the healthy subject population. Based on the results of prior studies at the 600 mg/day or lower doses [10, 12], the magnitude of induction observed in this study was likely near maximal or maximal and provided ample data for dose adjustment decisions.

The degree of induction observed in this study of CBZ and DTG was similar to that reported when DTG was co-administered with other potent CYP450 enzyme inducers such as tipranavir/ritonavir, efavirenz, and rifampin, which reduced DTG AUC(0-τ) in the range of 36 to 49 % and Cτ by 72 to 76 % [5, 6]. The substantial reduction in Cτ observed in these prior studies was considered clinically significant based on the DTG exposure–antiviral response relationship and observation that subjects receiving inducers in a phase III trial had lower antiviral response rates [14]. Therefore, the initial product labeling recommended an upward dose adjustment from 50 mg once daily to 50 mg twice daily when DTG is being co-administered with tipranavir/ritonavir, efavirenz, and rifampin in INI-naive subjects [1]. The present study showed that another strong enzyme inducer, CBZ, decreased DTG Cτ to a similar degree (~73 %) as tipranavir/ritonavir, efavirenz, and rifampin. Therefore, the same dose adjustment recommendation for DTG should be applied to CBZ. As with efavirenz and tipranavir/ritonavir, it is expected that DTG 50 mg twice daily co-administered with CBZ will demonstrate similar, if not higher, virologic response as that observed in subjects receiving DTG 50 mg once daily without these inducers. In INI-resistant subjects, where the exposures of the higher 50 mg twice-daily regimen are required for antiviral activity against resistant virus, co-administration with these strong enzyme inducers should be avoided [1].

Antiepileptic drugs are a diverse group of pharmacological agents and can generally be classified into two groups based on enzyme induction potential: enzyme-inducing and non-inducing AEDs [12]. Although unstudied, the enzyme-inducing AEDs such as phenytoin, oxcarbazepine, and phenobarbital are also expected to decrease DTG exposures and require DTG dose adjustment to 50 mg twice daily. Use of non-inducing AEDs such as gabapentin, lamotrigine, levetiracetam, tiagabine, and topiramate may be considered as an alternative to inducing drugs like CBZ to avoid DTG dose adjustment.

## Conclusion

This study evaluated the magnitude of a drug–drug interaction between CBZ, a known inducer of UGT1A1 and CYP3A, and DTG, a known substrate of UGT1A1 and CYP3A. As expected, co-administration of DTG and CBZ in healthy subjects resulted in a potentially clinically significant decrease in DTG AUC(0-τ), Cmax, and Cτ compared with DTG administered alone. Based on accumulated PK–pharmacodynamic relationship data regarding DTG and the currently approved dosing recommendation with other strong inducers, DTG dose adjustment to 50 mg twice daily is recommended when DTG is co-administered with CBZ.
